# Effect of NaF, SnF_**2**_, and TiF_**4**_ Toothpastes on Bovine Enamel and Dentin Erosion-Abrasion In Vitro

**DOI:** 10.1155/2012/134350

**Published:** 2012-11-08

**Authors:** Lívia Picchi Comar, Marina Franciscon Gomes, Naiana Ito, Priscila Aranda Salomão, Larissa Tercília Grizzo, Ana Carolina Magalhães

**Affiliations:** Department of Biological Sciences, Bauru School of Dentistry, University of Sao Paulo, Alameda Octávio Pinheiro Brisolla 9-75, 17012-901 Bauru, SP, Brazil

## Abstract

The aim of this study was to compare the effect of toothpastes containing TiF_4_, NaF, and SnF_2_ on tooth erosion-abrasion. Bovine enamel and dentin specimens were distributed into 10 groups (*n* = 12): experimental placebo toothpaste (no F); NaF (1450 ppm F); TiF_4_ (1450 ppm F); SnF_2_ (1450 ppm F); SnF_2_ (1100 ppm F) + NaF (350 ppm F); TiF_4_ (1100 ppm F) + NaF (350 ppm F); commercial toothpaste Pro-Health (SnF_2_—1100 ppm F + NaF—350 ppm F, Oral B); commercial toothpaste Crest (NaF—1.500 ppm F, Procter & Gamble); abrasion without toothpaste and only erosion. The erosion was performed 4 × 90 s/day (Sprite Zero). The toothpastes' slurries were applied and the specimens abraded using an electric toothbrush 2 × 15 s/day. Between the erosive and abrasive challenges, the specimens remained in artificial saliva. After 7 days, the tooth wear was evaluated using contact profilometry (**μ**m). The experimental toothpastes with NaF, TiF_4_, SnF_2_, and Pro-Health showed a significant reduction in enamel wear (between 42% and 54%). Pro-Health also significantly reduced the dentin wear. The toothpastes with SnF_2_/NaF and TiF_4_/NaF showed the best results in the reduction of enamel wear (62–70%) as well as TiF_4_, SnF_2_, SnF_2_/NaF, and TiF_4_/NaF for dentin wear (64–79%) (*P* < 0.05). Therefore, the experimental toothpastes containing both conventional and metal fluoride seem to be promising in reducing tooth wear.

## 1. Introduction

 Erosion is a tooth lesion caused by acids that is currently receiving a lot of attention from researchers and clinicians. This fact is due to the reports of its increasing prevalence [1] and also to the increasing knowledge about erosion etiology and diagnosis. The erosion lesion can be divided in two phases: erosion (in that there is only softening) and erosive wear (advanced phase with tooth surface loss) [2, 3].

In modern societies extrinsic acids are becoming the most important source of erosive attacks through the increased consumption of acid drinks (mainly soft drinks) [4]. The acid attack, in the advanced phase, leads to both wear and softening of the enamel surface. The remaining softened layer, especially for enamel, presents low wear resistance, thus rendering it more susceptible to the effects of mechanical forces, such as tooth-brushing abrasion [5, 6].

 Some studies have shown that the delay of 1 h of tooth-brushing after consuming acidic foods or drinks could minimize the deleterious effects on the eroded tooth surface [5–8]. However, this recommendation can be complicated in practice because it can interfere with the patient's routine. Therefore, new, more viable alternatives have been sought to prevent the effects of brushing on the eroded surface.

Tooth-brushing abrasion of eroded enamel and dentin is mainly influenced by the abrasivity of the toothpaste, and to a lesser extent by the toothbrush itself [9, 10]. Despite the possible abrasive effect of the toothpaste on eroded tooth surfaces, it plays an important role in maintaining general oral health (caries and periodontal diseases), and the daily fluoride exposure provides at least a slight protection against erosive demineralization from everyday exposure to acidic foods and drinks [11].

 Most commercial toothpastes have NaF as a fluoride agent. The beneficial effect of this conventional fluoride is associated with the formation of a CaF_2_-like precipitate on the tooth surface, which acts mainly as a fluoride reservoir and can partially behave as an acid-resistant layer [12, 13]. However, NaF presents limited beneficial effects compared to nonfluoridated toothpastes on the abrasion of eroded teeth [11, 14–19]. Based on this, recent studies have focused on fluoride compounds that might have a higher efficacy due to surface precipitation or incorporation of ions into demineralized tissue.

Accordingly, metal fluoride, such as SnF_2_ or TiF_4_, has been tested. SnF_2_ or SnCl_2_/NaF solution has shown a promising erosion-inhibiting effect both in vitro and in situ [20–22]; however, there are only limited results published about the effects of toothpastes containing SnF_2_ on erosion-abrasion [11, 23, 24]. In vitro and in situ studies have also demonstrated a considerable erosion-protection ability of the TiF_4_ solutions/varnish [25–30]. However, there is no study testing the effect of TiF_4_ toothpaste.

Therefore, the present in vitro study aimed to compare the effect of TiF_4_, NaF, and SnF_2_ toothpastes on enamel and dentin erosion and abrasion. The null hypothesis tested was that there is no significant difference among the performance of the fluoridated and nonfluoridated toothpastes to protect the eroded enamel and dentin against abrasion.

## 2. Materials and Methods

### 2.1. Preparation of the Specimens

One hundred and twenty enamel and dentin specimens (4 mm × 4 mm × 3 mm) were prepared from the labial surface of the bovine crown (one specimen/tooth, total: 120 teeth) and the labial/lingual outer surfaces of the cervical portion of bovine roots (two specimens/tooth, total: 60 teeth), respectively. The teeth were stored in 0.1% buffered thymol solution (pH 7.0) at 4°C. The specimens were cut using an ISOMET low-speed saw-cutting machine (Buehler Ltd., Lake Bluff, IL, USA) with two diamond disks (Extec Corp., Enfield, CT, USA), which were separated by a 4 mm thickness spacer. The specimens' surfaces were ground flat using water-cooled silicon carbide discs (320, 600, and 1200 grades of Al_2_O_3_ papers; Buehler, Lake Bluff, IL, USA) and polished using felt paper wet with diamond solution (1 *μ*m; Buehler). After the polish, the specimens were cleaned in an ultrasonic device with deionized water for 5 min. The removal of the cement from the root dentin by polishing was checked using a microscope (×40 magnification). Then, 2/3 of the outer surface (control, without treatment) specimens were covered with nail varnish in order to create control areas on both sides of a central band of enamel or dentin.

### 2.2. Experimental Groups, pH and F Analysis

Each 12 enamel and 12 dentin specimens were randomly allocated to each of 10 groups: placebo toothpaste without fluoride; experimental toothpaste with NaF (1450 ppm F); TiF_4_ (1450 ppm F); SnF_2_ (1450 ppm F); SnF_2_ (1100 ppm F) + NaF (350 ppm F); TiF_4_ (1100 ppm F) + NaF (350 ppm F); commercial toothpaste Pro-Health (SnF_2_—1100 ppm F + NaF 350 ppm F, pH 5.7, Oral B); commercial toothpaste Crest (NaF—1500 ppm F, pH 6.9, Procter & Gamble); abrasion without toothpaste; only erosion.

The experimental toothpastes were prepared by a Brazilian pharmacy (Farmácia Específica, Bauru, SP, Brazil). The experimental toothpastes presented the same composition except for the presence or not of fluoride and the type of fluoride salt.

The fluoride concentrations and pH of the toothpastes were further checked. The pH was analyzed in duplicate using an electrode and digital pH meter, after checking the standards (pH 4 and pH 7). The total fluoride (TF), soluble fluoride (SF), and ionic fluoride (IF) were evaluated following the study of Bardal et al. [31].

 To analyze the TF, 0.25 mL of 2 M HCl was added to 0.25 mL of the suspension of each toothpaste (0.5 g toothpaste/50 mL deionized water). This was kept at 45°C/1 h and 0.50 mL of 1 M NaOH and 1.0 mL of TISAB II were added. To assay SF and IF, after centrifugation, the supernatant was used, and the same steps described above were followed for SF. The analysis of IF was made by adding 0.25 mL of the supernatant to 1.0 mL of TISAB II, 0.5 mL of 1 M NaOH, and 0.25 mL of 2 M HCl. The analysis was done in duplicate using an ion-specific electrode (Orion 96-09) coupled to a potentiometer. The standard curve (0.5–4 ppm F) was prepared in triplicate using a standard fluoride solution (NaF, 100 ppm, Orion no. 940907). The potential (mV) was converted in *μ*g F using the standard curve with an *r* ≥ 0.99.

### 2.3. Treatment, Erosive, and Abrasive Challenges

All specimens were submitted to a 7-day erosive de- and remineralization cycling. Erosion was performed with a freshly opened bottle of the drink Sprite Zero (Coca-Cola Company Spal, Porto Real, RJ, Brazil, pH 2.6, 30 mL/specimen, unstirred, 25°C) four times daily for 90 s each. After each demineralization, the specimens were rinsed with deionized water (10 s) and transferred into artificial saliva (pH 6.8, 30 mL/specimens, unstirred, 25°C) for 2 h. After the last daily erosive treatment, the specimens were also stored in artificial saliva overnight. The artificial saliva was renewed daily and consisted of 0.2 mM glucose, 9.9 mM NaCl, 1.5 mM CaCl_2_·2H_2_O, 3 mM NH_4_Cl, 17 mM KCl, 2 mM NaSCN, 2.4 mM K_2_HPO_4_, 3.3 mM urea, 2.4 mM NaH_2_PO_4_, and 11 *μ*M ascorbic acid (pH 6.8) [32]. 

Twice a day (after the first and the last erosive challenges), the treatment was carried out with the slurries of the toothpastes (1 toothpaste: 3 deionized water, *v* = 0.5 mL/specimen) associated with abrasion, for 15 s each. The toothbrushes were fixed in the constructed device that allowed the heads of the toothbrushes to be aligned parallel to the surface of the specimens. The tooth-brushing head was weighted by a precision scale (Pesola, Switzerland) and the weight was converted to power (1 kg—9.80665 N, *F* = 1.5 N) [33]. After the tooth brushing, the specimens were rinsed in water for 10 s before being immersed in artificial saliva.

### 2.4. Profilometric Measurement

 Enamel and dentin wear (*μ*m) was quantitatively determined by a contact profilometer MSW 250/GD25 (Mahr Perthometer, Göttingen, Germany) after the experiment. For profilometric measurement, the nail varnish was carefully removed using a scalpel and acetone solution (1 : 1 water). During the measurement, the specimens were maintained in water (100% humidity) to avoid shrinkage of the dentin. The diamond stylus (2 *μ*m in diameter, angulation 90°) moved from the first reference to the exposed area and then over to the other reference area (2 mm long and 1.5 mm wide). Four profile measurements were performed for each specimen at intervals of 0.5 mm. The vertical distance between the horizontal line drawn on the reference areas and the horizontal line drawn on the experimental area was defined as tooth wear using the software (Software MahrSurf XT20, 2009). The values were averaged (*μ*m) and submitted to statistical analysis. The detection limit for the contact profilometry (MahrSurf XT20) is about 0.5 *μ*m of tooth wear. The standard deviation of repeated analysis (the reproducibility of the method) of a given sample, without removing it from the holder, was 0.2 *μ*m (mean wear of 2.8 *μ*m for enamel) and 0.5 *μ*m (mean wear of 3.4 *μ*m for dentin).

### 2.5. Statistical Analysis

The software GraphPad InStat (GraphPad Software, San Diego, CA, USA) was used. The assumptions of equality of variances and normal distribution of data were checked for all the variables tested, using the Bartlett and Kolmogorov-Smirnov tests, respectively. As the equality of variances was satisfied for enamel, the differences among the treatments were analyzed using ANOVA followed by Tukey's test. For dentin, the equality of variances was not satisfied, and the differences among the treatments were analyzed using Kruskall-Wallis followed by Dunn tests. The level of significance was set at 5%.

## 3. Results

Table 1 shows the mean values and standard deviation of the fluoride concentration and pH of the experimental toothpastes.

Table 2 shows the values of enamel and dentin wear. The enamel wear caused by erosion only was similar that caused by erosion plus abrasion without toothpaste. However, the enamel erosive wear was lower than the erosive and abrasive wear in the presence of the placebo toothpaste. The experimental toothpastes with NaF, SnF_2_, and TiF_4_, as well as the SnF_2_/NaF commercial toothpaste (Pro-Health), showed similar effect, reducing the enamel wear between 42% and 54% compared to the placebo toothpaste. The toothpastes containing SnF_2_/NaF and TiF_4_/NaF presented the best results regarding the enamel wear reduction (62–70% compared to the placebo toothpaste). Additionally, the combination of fluoride salts significantly reduced enamel wear compared to erosion only. The NaF-commercial toothpaste (Crest) was not significantly different than the placebo toothpaste. 

The dentin wear caused by erosion was also similar to that caused by erosion plus abrasion (with or without the placebo toothpaste). Both experimental and commercial NaF toothpastes were unable to reduce dentin wear. The commercial SnF_2_/NaF toothpaste (Pro-Health) significantly reduced dentin wear compared to the placebo, but not compared to tooth brushing without toothpaste or erosion only. The experimental toothpastes containing TiF_4_ and SnF_2_ significantly reduced dentin wear (64–73% compared to the placebo toothpaste). The toothpastes containing SnF_2_/NaF and TiF_4_/NaF also significantly reduced dentin wear (70–78% compared to the placebo toothpaste). Additionally, the metal fluoride alone or in combination in the experimental toothpastes significantly reduced dentin wear compared to erosion only.

## 4. Discussion

The present in vitro study assessed the effect of the experimental fluoridated toothpastes against tooth erosive and abrasive wear. The in vitro models are particularly well suited to experiments whose objective is to test a single process in isolation, where a more complex situation with many variables may confound the data. In this particular case, the present study followed the guidelines for the development and application of models for erosion research previously discussed by Shellis et al. [3]. We used bovine as a substitute for human teeth, due to the facility to obtain and prepare the samples from the former teeth. Although accurate data regarding the comparative properties of human and bovine hard dental tissue remain scarce, it is acceptable to use bovine as a substitute for human enamel and dentin [34, 35]. However, possible morphological and chemical property differences between bovine and human teeth substrates should be considered when the data are extrapolated to the clinical conditions. 

In the present study, samples from the outer surface of the cervical root region were submitted to the erosive and abrasive challenges, since this region is the first one exposed in patients suffering from periodontal recession. 

The contact profilometry is a method widely applied for analysis of tooth erosive/abrasive wear [36]. However, this method has some limitations, as it can damage the surface of the specimen, and in the case of dentin it can press or even penetrate into the demineralized organic layer in some extension, which is related to the moisture of the tissue. When it is important to consider the organic layer in the measurement of the dentin wear, the optical profilometry might be a more suitable method; however, when the loss of mineralized tissue is desirable, the organic layer should be removed prior to the use of contact profilometry [37, 38]. In the present study, to overcome partially this limitation, the dentin was kept in water during the measurement. 

Accordingly, the results of dentin wear in the present study must be interpreted taking into account the presence of the hydrated organic material. However, it should be kept in mind that the stylus may have penetrated into the demineralized organic layer, so some inaccuracy in the measurement might be expected. Considering this limitation, great variation on dentin wear was found. Therefore, any lack of effect of the treatments on dentin must be carefully interpreted [22].

The abrasive wear of eroded dental hard tissues is considered an adverse side effect of tooth brushing, and it is mainly determined by toothpaste abrasivity and pH [9, 10]. The present study confirmed this finding for enamel, in which the association between erosion and abrasion (in the presence of a placebo toothpaste) caused a higher wear than erosion only. However, this was not true in case of dentin, in accordance with Ganss et al. [39], who showed that significant effects of brushing were only found on the demineralized organic fraction (the toothbrush pressed the layer of collagen fibrils), but the mineral loss was unaffected.

In the presence of conventional fluoride (experimental NaF toothpaste), the enamel and dentin wear was reduced; however, the preventive effect was less pronounced than with the other fluoride salts, especially for dentin. The commercial version of NaF toothpaste (Crest) was unable to protect against tooth erosion and abrasion. The reason for the lack of effect of NaF commercial toothpaste might be related to its higher abrasivity. The fluoride effects might be partly masked by the toothpaste abrasivity, as shown by Moron et al. [40] and Hara et al. [41]. Generally, toothpastes with NaF have shown no or partial effect against tooth wear [11, 14, 15, 18, 19], which might be explained by the low acid resistance of the CaF_2_ layer, especially in case of in vitro models [13].

Following the results, the experimental TiF_4_ and SnF_2_ toothpastes, as well as the commercial Pro-Health toothpaste, were able to similarly reduce tooth wear, which was more pronounced in the case of dentin. This result showed that the effect of both metal fluorides is similar using this experimental model. Furthermore, even some differences in the abrasivity might be found between the experimental and commercial toothpastes containing SnF_2_, though this seems to be of less impact considering the presence of this metal fluoride.

One of the first studies conducted testing SnF_2_ toothpastes against tooth erosion showed that SnF_2_ toothpastes markedly reduced the dissolution of teeth in vivo, whereas NaF provided no protection [23]. Recently, Ganss et al. [11] and Faller et al. [42] have confirmed the superiority of SnF_2_ compared to NaF toothpastes to prevent dental erosion; however, for erosion-abrasion SnF_2_ toothpaste had no effect in vitro [11]. Contrary, Huysmans et al. [24] showed that SnF_2_-containing toothpastes significantly reduced erosive and abrasive wear compared to the NaF toothpaste using in situ protocol. Considering all findings, it seems that SnF_2_ is an effective fluoride salt to be incorporated into toothpaste for the prevention of tooth erosion and abrasion.

For dentin, the SnF_2_ has the advantage of reacting differently with the surface regardless of the presence of the collagen layer compared to the effect of NaF, which is dependent on the presence of the organic layer [43]. In cases in which the organic matrix is preserved, phosphoproteins might attract the tin ion, which is then retained in the organic matrix to some extent but also accumulates in the underlying mineralized tissue. In cases in which the organic matrix is removed, tin reacts with the mineral by precipitating different salts [44]. Furthermore, the inclusion of SnF_2_ into toothpaste could also minimize some side effects such as tooth staining and astringent taste, which were found for solutions containing high tin concentrations [21].

In respect to TiF_4_, there is no study testing the inclusion of this metal fluoride into toothpaste. Considering previous findings for the TiF_4_ solution and varnish, the positive effect found in the present study might be related to the precipitation and formation of an acid-resistant layer (glaze) by the interaction of titanium and phosphate from the tooth [45]. With respect to the interaction between TiF_4_ and dentin, it is not known if the reaction with a dentin surface might change in the presence or absence of a demineralized organic layer. Previous studies have shown that titanium tetrafluoride solution induces some coating on dentin surfaces, which partly covered dentinal tubules [46]. However, its protective potential did not exceed the efficacy of NaF or AmF [29, 46, 47].

 The present study was the first that associated a conventional and metal fluoride into experimental toothpastes, especially in the case of TiF_4_ and NaF. The toothpastes with SnF_2_/NaF and TiF_4_/NaF presented the best results in reducing tooth wear, even though they were similar to toothpastes containing only metal fluorides. Furthermore, the wear presented by the samples abraded using TiF_4_/NaF and SnF_2_/NaF toothpastes was also reduced compared to erosion only, which might be related to the surface precipitation (CaF_2_ plus metallic layers) or to the incorporation of ions (tin and Ti) into the demineralized tissue, making it more resistant not only to abrasion, but also to further erosive challenges.

The effect of adding metal and conventional fluorides into toothpaste should be confirmed using in situ models. The stability and the abrasivity (REA/RDA) of the experimental products, as well as the mechanism of action, should be further investigated. Based on the findings, it can be concluded that the association between conventional and metal fluorides in toothpastes might be a promising strategy to reduce tooth wear provoked by the synergic effect of erosion and abrasion in vitro.

## Figures and Tables

**Table 1:** Mean (±sd) of fluoride concentration (ppm or *μ*g/g) and pH of the experimental toothpastes.

Toothpastes	pH	Ionic fluoride	Soluble fluoride	Total fluoride
Placebo	5.6	31.1 ± 6.2	52.5 ± 13.9	48.6 ± 5.6
NaF 1450 ppm F	6.0	1324.5 ± 70.4	1323.3 ± 111.0	1324.5 ± 70.4
TiF_4 _1450 ppm F	3.8	1403.9 ± 31.4	1606.2 ± 31.8	1443.2 ± 8.1
SnF_2_ 1450 ppm F	3.7	1307.2 ± 14.6	1406.5 ± 3.9	1323.6 ± 66.6
SnF_2_ (1100 ppm F) + NaF (350 ppm F)	3.9	1474.9 ± 12.4	1522.7 ± 46.9	1567.0 ± 109.5
TiF_4_ (1100 ppm F) + NaF (350 ppm F)	4.4	1275.3 ± 57.1	1203.6 ± 76.1	1207.3 ± 81.1

**Table 2:** The erosion-abrasion wear in the presence of the different toothpastes (*μ*m).

Group	Enamel wear mean (±sd)	Dentin wear median (min–max)
Placebo	5.0 ± 0.9^a^	10.8 (7.5–13.5)^a^
NaF 1450 ppm F	2.9 ± 0.7^c^	5.6 (3.6–9.1)^abcd^
TiF_4 _1450 ppm F	2.3 ± 0.7^cd^	2.9 (1.3–6.2)^de^
SnF_2_ 1450 ppm F	2.8 ± 1.1^c^	3.9 (2.1–6.9)^cde^
SnF_2_ (1100 ppm F) + NaF (350 ppm F)	1.7 ± 0.7^d^	2.3 (1.2–3.7)^e^
TiF_4_ (1100 ppm F) + NaF (350 ppm F)	1.5 ± 0.4^d^	3.2 (1.0–5.8)^de^
Pro-Health (Oral B)	2.9 ± 1.0^c^	4.6 (3.0–7.9)^bcde^
Crest (Procter & Gamble)	4.1 ± 0.9^ab^	7.1 (4.3–10.0)^abc^
Without toothpaste	4.1 ± 0.9^ab^	7.7 (6.1–11.4)^ab^
Only erosion	3.1 ± 0.9^bc^	9.7 (4.7–12.9)^ab^

Different letters indicate statistically significant differences among the groups (comparison within the same column, *P* < 0.0001).
